# The effect of cyclic knee motion on the elongation of four-strand hamstring autograft in anterior cruciate ligament reconstruction: an in-situ pilot study

**DOI:** 10.1186/s12891-019-2699-5

**Published:** 2019-07-09

**Authors:** Dong Jiang, Ying-fang Ao, Chen Jiao, Qin-wei Guo, Xing Xie, Feng Zhao, Nan Li, Xiao-xiao Wang, Yue-lin Hu

**Affiliations:** 1 Institute of Sports Medicine, Peking University Third Hospital, Beijing Key Laboratory of Sports Injuries, Beijing, 100191 China; 2 Research Center of Clinical Epidemiology, Peking University Third Hospital, No.49 North Garden Road, Haidian, Beijing, 100191 China

**Keywords:** Anterior cruciate ligament reconstruction, Arthroscopy, Cyclic knee motion, Pretension, Graft lengthening

## Abstract

**Background:**

Pretension of the viscoelastic graft by cyclic knee motion has been confirmed to decrease the graft creep and improve the outcome of anterior cruciate ligament (ACL) reconstruction. The purpose of the present study was to investigate the effect of cyclic knee motion on the elongation of the four-strand hamstring tendon autograft in situ and to explore the stable level cycle, in which the tendon length achieved a stable level.

**Methods:**

The study was performed with 53 consecutive patients undergoing transtibial ACL reconstruction with four strand hamstring tendon from Aug 2013 to Apr 2015. 43 males and 10 females were included with mean age of 29 ± 10 years. The pretension of the tendons was operated by cyclical knee motion ranging from 0 to 110°after the femoral fixation with Endo-button. The tendon length after 10, 20, 30 and 40 cycles was measured respectively and compared by repeated measure ANOVA. Then multivariate logistic regression was used to investigate the effect of the patients’ parameters (age, gender, height, body weight, tendon length, etc.) on the elongation of the graft and the stable level cycle.

**Results:**

The mean lengthening of the graft at 10, 20, 30 and 40 times was 3.0 ± 1.4 mm, 4.3 ± 1.5 mm, 4.8 ± 1.7 mm and 4.8 ± 1.8 mm respectively. No significant correlation was found between the elongation and the patients’ parameters. There was significant difference of the tendon length from 0 to 30 cycles (F = 264.8, df = 1.95, *p*<0.001). However, the tendon length achieved a stable level after 30 cycles and the median elongation from 30 cycles to 40 cycles was 0 (0–1) mm with no significant difference (F = 2.039, *p* = 0.159). The male and female tendon length achieved to a stable level at 20 cycles and 30 cycles respectively but with no significant difference (*p* = 0.074).

**Conclusions:**

The four-strand hamstring tendon was elongated after cyclic knee motion and the elongation achieved a stable level after 30 cycles for the transtibial technique. Both of the tendon elongation and the stable level cycle were not correlated with patients’ gender, age, preoperative duration, graft diameter and length.

## Background

Anterior cruciate ligament (ACL) rupture is one of the most common sports injuries of the knee joint, leading to pain, swelling, giving way and instability [1]. ACL reconstruction restores joint stability but the outcome was affected by many factors, of which the initial strength of the graft is important [2]. Several studies showed that the initial graft tension would influence the results of ACL reconstruction [3–5]. In fact, the viscoelastic creep is associated with collagenous soft tissues under sustained tensile load and the graft elongation that occurs after fixation is a key factor in mechanical failure [6]. According to literatures, pretension of the viscoelastic graft before fixation could decrease the amount of elongation and the pretension treated graft has been shown to have significantly greater stiffness than the control group [7]. Thus applying tension to the ACL graft has been a technically important aspect of ACL reconstruction to prevent post-implantation graft creep [8, 9].

For the graft pretension, it could be operated by applying a cyclic or static load to the graft during fixation. The repeated flexion and extension of the knee joint has been a simple and practical procedure for pretension [10]. Lee CH et al. also found that the intra-articular pretension grafts has significantly less displacement than grafts subjected to other 2 extra-articular pretension methods in an animal experimental study [11]. However, the previous studies were mainly focused on the bone-patellar tendon-bone grafts (BPTB) [4, 12, 13] or the semitendinosus and gracilis plus polysters [3] or fixed with interference screw [14]. As a widely used ACL reconstruction procedure, four strand hamstring autograft with Endo-button as femoral fixation has been rarely studied about its pretension in situ. Thus it’s necessary to provide evidence for the best approach of pretention, which also helps in creating new guidelines.

In the present study, 53 ACL reconstruction patients were included and the pretension of the grafts was operated by cyclic knee motion ranging from 0 to 110° after the femoral fixation with Endo-Button. The purpose of this study is (1) to investigate the lengthening of the four-strand hamstring tendon autograft after cyclic knee motion during ACL reconstruction and establish the optimal number of cycles; (2) to analyze the factors related to the tendon elongation and the stable level cycle, in which the tendon length achieved a stable level. It was hypothesized that the length of the tendon would increase and achieve a stable level after certain cyclic knee motion. The graft lengthening and the stable level cycle might be related to patients’ parameters including age, gender, injured side, height, body weight, pre-operative duration, bone tunnel diameter, tendon length and Endo-button loop length.

## Methods

### Patients

This study was approved by the board of research ethics in our hospital (IRB00006761–2011097). The patients underwent arthroscopic ACL reconstructions in our institute from August 2013 to April 2015. The inclusion criteria included: transtibial bone tunnel positioning technique; four-strand hamstring tendon (semitendinosus and gracilis, STG) autograft with a sufficient length to make sure that the distal graft protruding the distal tibial tunnel outlet is more than 2.5 cm; Endo-button as the femoral side fixation; being done by the same surgeon. The exclusion criteria included contaminant other ligament injuries.

The patients were continuously enrolled during the study and there was a total of 53 patients meeting the inclusion criteria including 43 males and 10 females with 24 left knees and 29 right knees. The mean age was 29.4 ± 10.2 years. The preoperative duration from the initial injury to the surgery was 48 (0.1–240) months. The ACL rupture was diagnosed by Lachman test and MRI and confirmed by arthroscopic exploration.

### Surgical technique and measurement

The patient’s informed consent was obtained before surgery. The surgical procedures have been described in our previous study [15]. Under general or spinal anaesthesia, the patients were positioned supine on the operating table. Standard diagnostic arthroscopy was performed to confirm ACL rupture and other injuries. Then the autogenous STG tendons were harvested through a 2–3 cm oblique incision. After tendons were cleaned of muscle tissue, the length of semitendinosus tendon (ST) and gracilis tendon (GT) and the diameter of quadrupled STG were measured. Then ends of each tendon were whipstitched for 2.5 cm with No.5 Ethibond sutures (Ethicon, Somerville, NJ) at the preparation board and then pretensioned for 15 min to reduce the influence between tendon and suture.

In order to directly measure the total length of the femoral and tibial tunnels, the transtibial technique was used for ACL reconstruction. The exact tibial tunnel placement was at the intersection of the free margin extension of the lateral meniscus anterior horn and the lateral slope of the medial intercondylar spine. In this way, the tibial tunnel was placed at the second part from anterior by Harner’s quadrant evaluation [16]. The anterior edge of the PCL fibers was also taken as reference to avoid individual variability. The 2 mm metallic Kirschner wire was inserted with the help of a tibial guide (Smith & Nephew Endoscopy, Andover MA) transferred to 45° marker and 30° medially away from the tibia tubercle. Through trans-tibia approach with a 5-6 mm femoral guide (Smith & Nephew Endoscopy, Andover MA), the femoral tunnel was positioned at the most proximal high deep point of the lateral intercondylar cartilage edge, a little bit inferior to the site identified in the over-the-top technique. Following the guidance of Kirschner wire, the bone tunnel was drilled with 4.5 mm and 7-8 mm drill according to the graft diameter.

With the knee flexed at 90°, the length of the femoral bone tunnel was measured to determine the Endo-button (Smith & Nephew Endoscopy, Andover MA) loop length (making graft in the femoral bone tunnel ≥2 cm). The mean Endo-button loop length was 25(15–35) mm. Then the distance between the proximal outlet of the femoral tunnel and the distal outlet of the tibial tunnel (ie total bone tunnel length) was measured. Thus the length of the intra-body tendon (IBT) was calculated by the total bone tunnel length minus Endo-button loop length. The IBT actually included the tendon of both intra-bone tunnel and intra-articular parts.

**Fig. 1:**
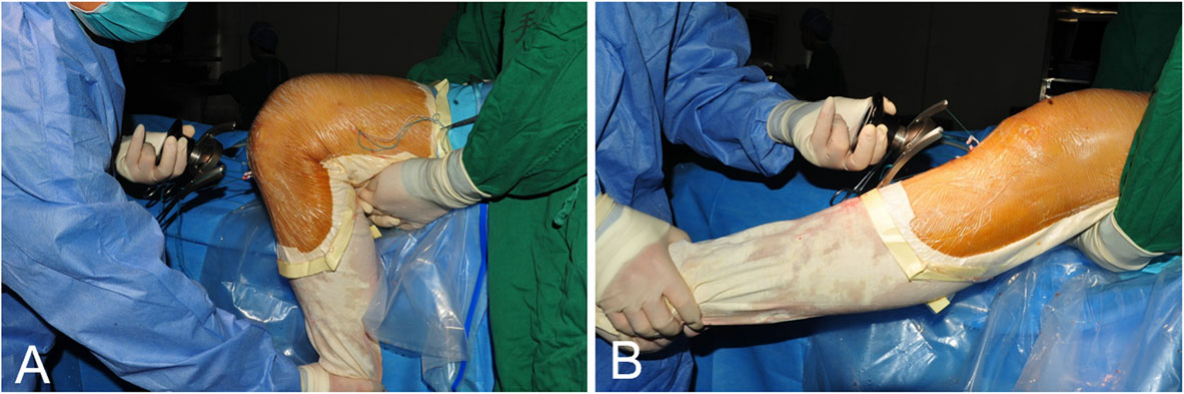
Graft pretension procedure. The graft was tightened by a tensometer with the tension of 15 lb. Then the pretension was operated by repeated flexing and extending the knee joint between 0°(**b**) and 110° (**a**)

The STG autografts were quadrupled at the Endo-button loop and pulled into the bone tunnel. The Endo-button was confirmed to fasten at the femoral cortical surface by arthroscopic lateral gutter visualization. Then the knee was placed at flexion 90° and the first mark was made at the 10 cm distal to the tibial tunnel outlet. The graft was tightened by a tensometer (*DePuy* Mitek Inc., Raynham, MA) and then the pretension was operated by repeated flexing and extending the knee joint between 0 and 110° with the tension maintained at 20 lb. by the tensometer (Fig. 1). After 10, 20, 30 and 40 cycles, the length from the 10 cm mark to the distal tibial tunnel outlet was measured with vernier caliper respectively. Then the INTRAFIX absorbable screw system (*DePuy* Mitek Inc., Raynham, MA) was used for tibial fixation.

### Statistics

All statistical analyses were conducted using IBM SPSS Statistics software, version 22.0 (IBM Corporation, Armonk, NY). The tendon length at different flexion cycles was compared by repeated measure ANOVA. The sphericity of the data was evaluated by Mauchly sphericity test and the Greenhouse-Geisser correction was used for the data not meeting the sphericity. The difference between the two groups were compared by t test, nonparametric test or chi-square test. Univariate correlation was operated by Spearman correlation analysis and the multivariate logistic regression was used to investigate the related factors of the stable level cycle. *P* < 0.05 was considered as statistically significant.

## Results

**Fig. 2:**
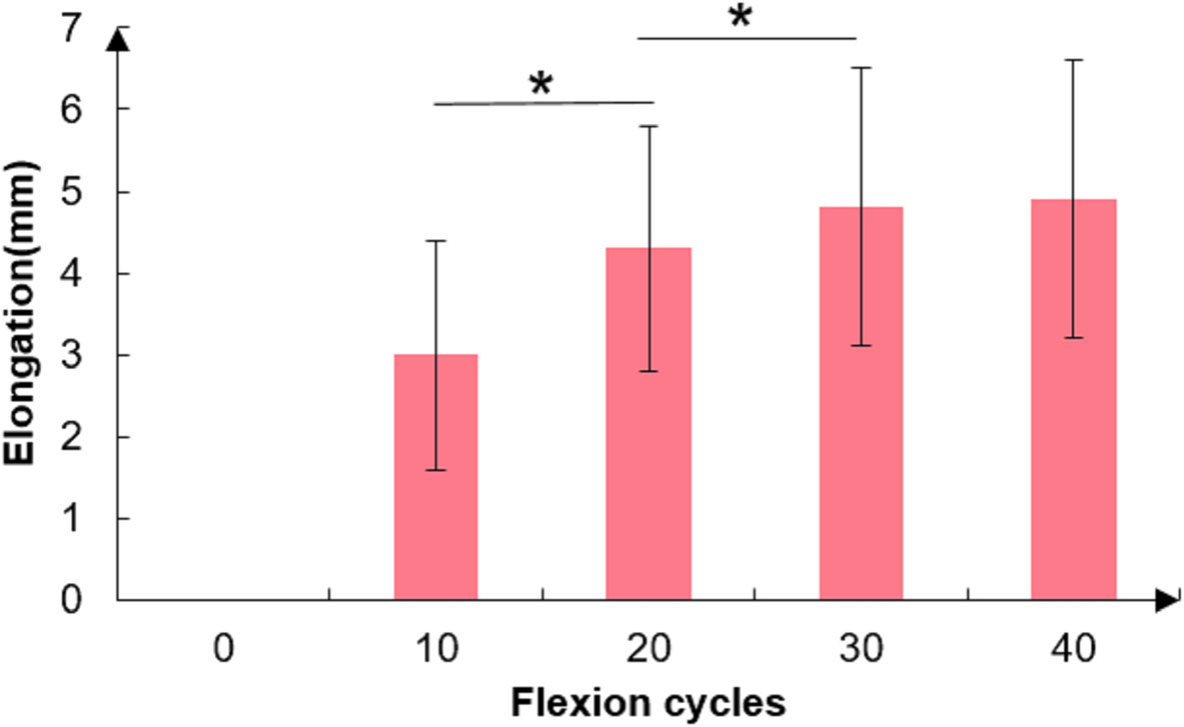
Relationship of elongation and the flexion cycle

The mean length of ST, GT and IBT was 272 ± 32 mm, 246 ± 32 mm and 85 ± 8 mm respectively. The mean diameter of the four-strand STG autograft was 7.8 ± 0.4 mm. The elongation of the 4 strand STG graft was shown in Table 1 and Fig. 2. The relative lengthening percentage to the ITT was also calculated to exclude the influence of tendon length. The repeated measure ANOVA was used to compare the elongation at each point (including the initial length). Mauchly sphericity test indicated that the data did not meet the sphericity (w = 0.152, *p* < 0.001). Using Greenhouse-Geisser correction, significant difference was shown for the tendon elongation (F = 264.8, df = 1.95, *P*<0.001). According to the comparison of intra-cyclic elongation, there was significant difference for the 0 vs 10 cycles (F = 243.176, *P* < 0.001), 10 vs 20 cycles (F = 143.307, *P* < 0.001) and 20 vs 30 cycles (F = 43.505, *P* < 0.001). However, the median elongation from 30 cycles to 40 cycles was only 0(0–1) mm with no significant difference (*P* > 0.05). In fact, the tendon lengthening occurred in only 2 patients (3.8%) after 30 cycles (Table 1).

**Table 1:** The elongation of 4 strand STG graft after repeated knee flexion

	10 cycles	20 cycles	30 cycles	40 cycles
Elongation (mm, %)^a^				
Mean	3.00(3.6%)	4.32(5.2%)	4.81(5.8%)	4.85(5.8%)
SD	1.40(1.7%)	1.52(1.9%)	1.72(2.1%)	1.77(2.2%)
Median	3.0(3.4%)	4.1(4.7%)	5.2(5.6%)	5.2(5.6%)
Min	1.0(1.1%)	2.1(2.0%)	2.2(2.6%)	2.2(2.6%)
Max	7.2(8.8%)	9.0(11.3%)	9.8(12.5%)	10.0(12.5%)
Elongation cases (%)^b^	53(100%)	46(86.8%)	25(27.2%)	2(3.8%)

^a^The elongation and the relative percentages to the IBT

^b^The number and percentage of cases whose length changes after each 10 cycles

The correlation of the tendon elongation and the patients’ parameters at each point was analyzed in Table 2 and no significant correlation was found (*P* > 0.05). In terms of gender (Z = -1.189, *P* = 0.234) and injured side (Z = -0.897, *P* = 0.370), there was no significant correlation either.

**Table 2:** Correlation analysis between graft elongation and patients’ parameters

	10 cycles		20 cycles		30 cycles		40 cycles	
	CC	*P*	CC	*P*	CC	*P*	CC	*P*
Age	0.020	0.885	0.028	0.840	0.030	0.829	0.031	0.823
Height	0.014	0.919	0.07	0.616	0.057	0.688	0.075	0.593
Body weight	0.081	0.562	0.056	0.689	0.036	0.795	0.047	0.740
Pre-op duration	0.161	0.251	0.142	0.311	0.174	0.212	0.181	0.195
Graft diameter	−0.117	0.404	0.002	0.991	−0.011	0.936	0	0.982
ST length	−0.023	0.868	−0.040	0.776	−0.134	0.337	−0.13	0.364
GT length	−0.006	0.966	−0.030	0.832	−0.108	0.441	−0.11	0.434
IBT length	0.037	0.794	0.012	0.933	−0.001	0.993	−0.02	0.862
Loop length	0.061	0.662	−0.039	0.784	−0.014	0.921	−0.01	0.962

*CC* correlation coefficient, *ST* semiteninous tendon, *GT* gracilis tendon, *IBT* intra-body tendon

As shown in Table 1, the graft length achieved to a stable level (ie. stable length) with a mean of 25+/−6 cycles, in which the tendon length achieved the stable level. Since the vast majority of patients (51 cases, 96.4%) achieved to the stable length at 20 or 30 cycles, the parameters were compared between the two groups (28 cases for 20 cycles and 23 cases for 30 cycles) to investigate the related factors of the stable level cycle (Table 3). The results showed no significant difference between the two groups (*P* > 0.05). Then the correlation was also analyzed by multivariate logistic regression (Table 4). Similar to univariate analysis, no significant correlation was found between the stable level cycle and the patients’ parameters. However, the male and female tendon length achieved to a stable level at 20 cycles and 30 cycles respectively but with no significant difference (*P* = 0.074).

**Table 3:** Univariate analysis of stable level cycle with patients’ parameters

	Stable level cycle = 20(*n* = 28)	Stable level cycle = 30 (*n* = 23)	t/z/χ^2^	*P*
Age	29.8 ± 11.4	28.5 ± 8.8	0.45	0.655
Height(cm)	174.0 ± 8.6	173.7 ± 9.0	0.137	0.891
Body weight(kg)	77.3 ± 13.5	75.6 ± 15.6	0.414	0.681
ST length(cm)	27.7 ± 3.5	26.5 ± 2.8	1.381	0.173
GT length(cm)	25.1 ± 3.5	24.0 ± 2.7	1.325	0.191
IBT length(mm)	85.0 ± 8.4	84.3 ± 7.6	0.325	0.746
Endo-button loop(mm)	23.0 ± 4.2	23.5 ± 4.9	−0.35	0.728
Pre-op duration(month)	7.5(0.1–144.0)	6.0(0.5–240.0)	−0.142	0.887
Male (%)	24(85.7%)	17(73.9%)	1.094	0.296
Left side (%)	14(50.0%)	10(43.5%)	0.216	0.642
Graft diameter (%)	5(17.9%)	5(21.7%)	0.121	0.728

*ST* semiteninous tendon, *GT* gracilis tendon, *IBT* intra-body tendon

**Table 4:** Multivariate logistic regression of stable level cycle and patients’ parameters

	B	S.E.	*P*	OR	95% OR CI	
					lower limit	uper limit
Age	.014	.038	.704	1.015	.942	1.093
Height	.148	.096	.124	1.159	.960	1.400
Body weight	−.011	.035	.744	.989	.924	1.058
ST length	−.143	.216	.507	.866	.567	1.323
GT length	−.191	.208	.358	.826	.550	1.242
IBT length	.015	.059	.803	1.015	.904	1.139
Endobutton loop	.018	.092	.843	1.018	.850	1.219
Pre-op duration	.004	.008	.583	1.004	.989	1.020
Gender (male)	2.277	1.273	.074	9.745	.804	118.076
Side (left)	.491	.699	.483	1.633	.415	6.429
Graft diameter	−.826	1.115	.459	.438	.049	3.894
Constant	−12.940	12.469	.299	.000		

*OR* odds ratio, *CI* confidence interval, *ST* semiteninous tendon, *GT* gracilis tendon, *IBT* intra-body tendon

**Fig. 3:**
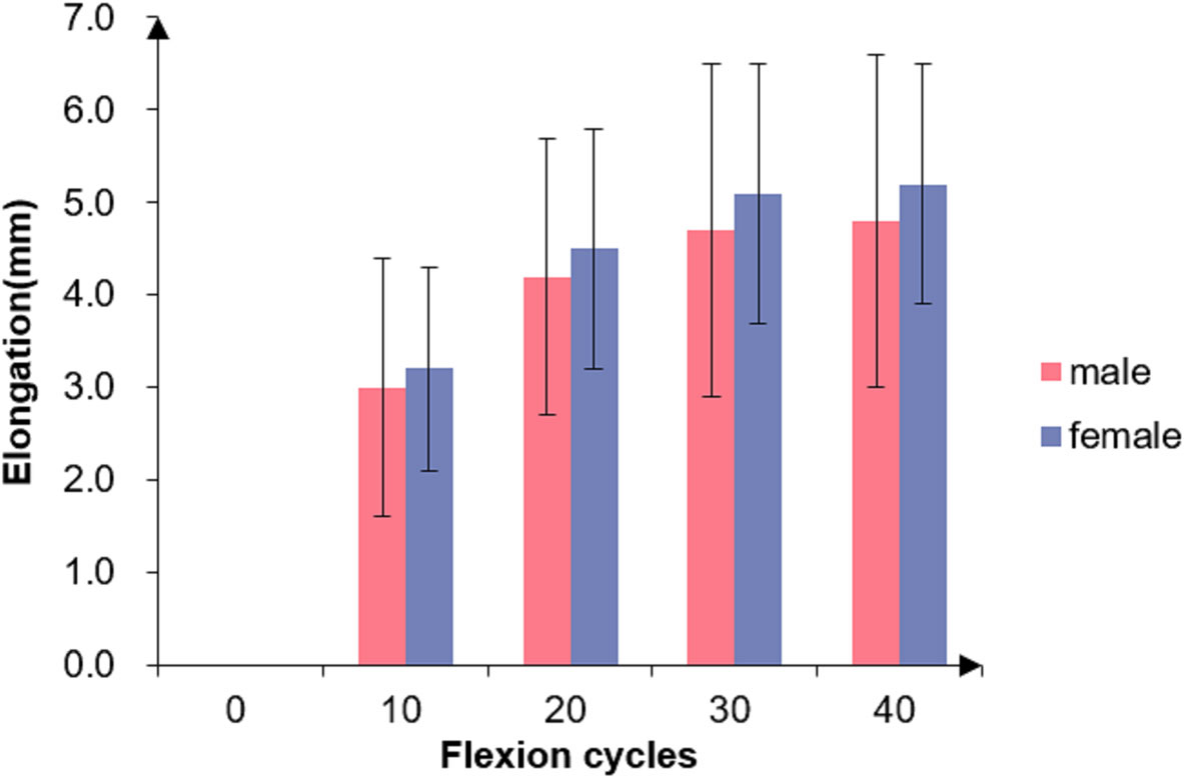
Graft elongation of different gender patients

In terms of the effect of gender, the tendon was lengthened by repeated knee motion in both male and female patients (Fig. 3) with a significant difference of intra-cyclic lengthening (F = 197.981, df = 1.655, *P*<0.001). There was a similar tendency for the tendon lengthening among different sex groups. Although the female tendon was lengthened more than the male’s, no significant difference was found at each point (F = 0.272, *P* = 0.605) (Fig. 3).

## Discussion

This study investigated the effect of cyclic knee motion on the elongation of the four-strand hamstring tendon autograft with suspensory fixation during ACL reconstruction. The graft was indeed lengthened after pretension by the cyclic knee motion, but no significant correlation was found between the elongation and the patients’ parameters. The tendon length achieved a stable level after 30 cycles.

The 4 strand STG autograft of the present study was significantly lengthened with repeated knee flexion and extension, which might be associated with postoperative mechanical failure [6]. Beynnon BD et al. [12] compared the 5-year follow-up results with or without pretension in ACL reconstruction and found that the graft elongation values produced by flexion of the knee that are outside the limits of the ACL result in significant increases in anterior knee laxity at long-term follow-up, while grafts with elongation values similar to the normal anterior cruciate ligament do not. Yasuda et al. [3] also demonstrated that relatively high initial tension (up to 80 N) reduced the postoperative anterior laxity of the knee joint after anterior cruciate ligament reconstruction using the doubled autogenous hamstring tendons connected in series with polyester tapes. Thus applying tension to the ACL graft has been a technically important aspect of ACL reconstruction to prevent postimplantation graft creep [8, 9]. However, the effect of the pretension on the outcome of the ACL reconstruction could not be concluded from the present study and a long-term comparative research was needed in the future.

Four-strand hamstring autograft with Endo-button has been a widely used ACL reconstruction procedure in recent years. Biomechanical studies found that equally tensioned four-strand hamstring-tendon grafts have higher initial tensile properties than those in the original and BPTB graft [17, 18]. However, compared to direct bone fixation tendon interface, such as interference screw, the loop of the cortical button fixation device might easily produce wiper effect or elastic cord effect leading to postoperative bone tunnel widening and ligamentous laxity [19–21]. Therefore, for the Endo-button hamstring ACL reconstruction, the graft pretension becomes more important. Brand et al. found that the Endo-button fixation is not as stiff as either of the femoral interference fixation options, but the addition of more than 20 loading cycles could remove laxity from the graft fixation-graft cruciate ligament complex and improve its stiffness [19]. Based on the result of the present study, after 30 cycled knee flexion and extension, the graft could be fully pretensioned.

In the present study, the elongation of the 4 strand STG graft achieved to 4.8 ± 1.8 mm after 40 cycles, which seemed less than other studies with the same graft. Kawano CT et al. [14] pretentioned the grafts of the STG in situ with range of flexion and extension of 0–110°. The lengthening was 3.57 mm/3.97 mm with ten, 6.30 mm/7.03 mm with 25, and 6.83 mm/7.7 mm with 50 cycles in the interference and transcondylar groups, respectively. The difference might be explained by the different femoral fixation, patient race and composition. It should be noted that the source of the elongation was still unclear in spite that the elongation value has been investigated. Höher J et al. indicated that the interfaces between the different materials such as tendon to tape, tape to button, tendon to suture, and suture to screw are the primary sites of constructed elongation [22]. In the present study, the distal graft protruding the tibial tunnel outlet was more than 2.5 cm, which excluded the effect of the suture. Thus the elongation of graft might mainly come from the tendon to tape and the tendon itself.

Although the pretension may increase the initial tension of the graft, excessive pretension may also lead to postoperative articular cartilage injury, long-term instability and delayed recovery [23]. Thus it is essential to determine the stable level cycle of flexion and extension. Boylan et al. found that the tension of hamstring tendon fell 50.2% after 1000 cycled knee flexion and extension between 0 and 90 ° and the anterior laxity significantly decreased [24]. However, it seemed with poor operability to perform 1000 cycles in ACL reconstruction. Arnold et al. [25] found that only 50% of the initial tension applied to a patellar tendon graft remained after 1500 cycles of knee flexion, with the most rapid decrease occurring in the first 100 cycles. Kawano CT etc. [14] also found that the lengthening from 25 times to 50 times were only 0.53 mm and 0.64 mm respectively, thus the authors recommend 25 cycles of intra-operative flexions to be appropriate. Those results indicated that most of the tendon elongation occurred at the early cycles. Our study showed the similar result. More elongation of the graft was found at the first 30 cycles and there was only 2 patients (3.8%) whose tendon lengthened after 30 cycles with median elongation of 0(0–1) mm. Thus it could be believed that 30 cycles achieved a relatively stable elongation of the 4 strand STG graft with the Endo-button as femoral fixation.

In terms of related factor affecting the tendon elongation, this study indicated no significant correlation of the tendon elongation with the patient’s parameters. Meanwhile, no correlation was found in terms of the stable level cycle. However, Kawano CT found that the greater the length of the graft, the greater the lengthening on pretensioning throughout the substance; the shorter the length, the earlier the end of the lengthening was achieved [14]. The reason might be due to the difference in the femoral fixation. In addition, there are only 53 patients included in this study, out of which 43 are males and 10 are females. Maybe the insufficient sample size leads to the results that there are no significant correlation between the tendon elongation and the stable level cycle with patients’ parameters.

The pretension methods in ACL reconstruction included static and dynamic cycle pretension. Other than the static pretension of 60-80 N for 10–20 min, which was usually used for graft pretention, the method of repeated knee flexion and extension was performed in the present study. Pilia M et al. [7] confirmed that a simple pull up to 80 N before fixation does not impart sufficient tension to a graft to prevent it from failing and they recommend precondition or pretension the tendons before final tibial fixation to achieve greater retained tension in the graft after placement. Lee CH et al. [11] compared different commonly used pretension techniques for ACL reconstruction (manual, extra-, and intra-articular pretension) and the results suggested that the intra-articular pretension of the graft before final fixation can significantly minimize graft elongation at time 0 compared with the other methods. Thus they recommended the intra-articular or in vivo pretension of the graft to minimize the graft elongation in the early period of rehabilitation after ACL reconstruction. Meanwhile, the cyclic pretention of the graft was operated in the bone tunnel after femoral fixation to ensure the optimum level of graft tension for the knee joint [14]. Furthermore, compared to tensioning on the graft preparation board for 20–30 min [6, 26], the cycled motion pretension seemed relatively more practical to save operative time. On the other hand, Nishizawa Y et al. [27] found that the intra-articular graft tension was significantly lower than the applied tension from the outside of the joint, even after cyclic loading and pretensioning. However, the author also stated that it is difficult to evaluate the intra-articular graft tension precisely on the basis of the extra-articular tension at time zero in ACL reconstruction.

The present in-situ study evaluated and highlighted the effect of cycled knee flexion on the elongation of four strand STG with Endo-button. Several previous similar studies were performed with BPTB [4, 12, 13], fixation with interference screws [14] or in cadaveric [6, 10, 17, 19, 28]. The biomechanics of BPTB was significantly different with that of the STG. The cadaveric results could not be directly used for clinical practice. The actual elongation might be better reflected though an in-situ study by live tendon graft in the live bone tunnels. The related factors of the graft have been rarely studied, which was important for more accurate pretension to each patient.

There are still some limitations for the present study. First, the present study is just to measure the tendon lengthening at surgery, the finding of the best cycles in the present study should be proved effective with follow-up. In addition to the graft inherent viscoelasticity, the reconstructed graft degeneration, fixation, bone tunnel widening and the postoperative rehabilitation may also affect the tension of the graft [29]. Second, more male patients and those with diameter of 8 mm were included in the study. The uneven proportion of gender and graft diameter might affect the result. In addition, the ACL reconstruction was performed with transtibial technique for the measurement needs of this study, the appropriate number of flex/ext. cycles in anteromedial portal femoral tunnel positioning technique is still unclear. Despite the fact that the transtibial technique has become less popular for ACL reconstruction, clinically important outcomes are actually comparable to literature figures for all the reported drilling techniques [30]. Furthermore, as the graft preparation board has been widely used nowadays, the comparison of the elongation and the clinical outcome between the static and the cyclic pretension might be also needed.

## Conclusions

The present in-situ pilot study indicated that the four-strand hamstring tendon was elongated after cyclic knee motion and the elongation achieved a stable level after 30 cycles for transtibial technique. Both of the tendon elongation and the stable level cycle were not correlated with patients’ gender, age, preoperative duration, graft diameter and length. Thus at ACL reconstruction, the four-strand hamstring tendon could achieve a relatively stable length by 30 cycles of repeated knee motions.

## Data Availability

The raw data available upon reasonable request from the corresponding author (Hu YL).

## Abbreviations

ACL: anterior cruciate ligament
CC: correlation coefficient
CI: confidence interval
GT: gracilis tendon
ITT: intra-tunnel tendon
OR: odds ratio
ST: semiteninous tendon

## References

[1] Filbay SR, Culvenor AG, Ackerman IN, Russell TG, Crossley KM (2015). Quality of life in anterior cruciate ligament-deficient individuals: a systematic review and meta-analysis. Br J Sports Med.

[2] Lynch TS, Parker RD, Patel RM (2015). The impact of the multicenter Orthopaedic outcomes network (MOON) research on anterior cruciate ligament reconstruction and Orthopaedic practice. J Am Acad Orthop Surg.

[3] Yasuda K, Tsujino J, Tanabe Y, Kaneda K (1997). Effects of initial graft tension on clinical outcome after anterior cruciate ligament reconstruction. Autogenous doubled hamstring tendons connected in series with polyester tapes. Am J Sports Med.

[4] van Kampen A, Wymenga AB, van der Heide HJ, Bakens HJ (1998). The effect of different graft tensioning in anterior cruciate ligament reconstruction: a prospective randomized study. Arthroscopy..

[5] Nicholas SJ, D'Amato MJ, Mullaney MJ, Tyler TF, Kolstad K, McHugh MP (2004). A prospectively randomized double-blind study on the effect of initial graft tension on knee stability after anterior cruciate ligament reconstruction. Am J Sports Med.

[6] Piedade SR, Dal Fabbro IM, Mischan MM (2006). Cyclic-loading of the human gracilis and semitendinosus muscle tendons: study of young adult cadavers. Artif Organs.

[7] Pilia M, Murray M, Guda T, Heckman M, Appleford M (2015). Pretensioning of soft tissue grafts in anterior cruciate ligament reconstruction. Orthopedics..

[8] Howard ME, Cawley PW, Losse GM, Johnston RB (1996). Bone-patellar tendon-bone grafts for anterior cruciate ligament reconstruction: the effects of graft pretensioning. Arthroscopy..

[9] Arneja S, McConkey MO, Mulpuri K (2009). Graft tensioning in anterior cruciate ligament reconstruction: a systematic review of randomized controlled trials. Arthroscopy..

[10] Mae T, Shino K, Nakata K, Toritsuka Y, Otsubo H, Fujie H (2008). Optimization of graft fixation at the time of anterior cruciate ligament reconstruction. Part I: effect of initial tension. Am J Sports Med.

[11] Lee CH, Huang GS, Chao KH, Wu SS, Chen Q (2005). Differential pretensions of a flexor tendon graft for anterior cruciate ligament reconstruction: a biomechanical comparison in a porcine knee model. Arthroscopy..

[12] Beynnon BD, Uh BS, Johnson RJ, Fleming BC, Renstrom PA, Nichols CE (2001). The elongation behavior of the anterior cruciate ligament graft in vivo. A long-term follow-up study. Am J Sports Med.

[13] Yoshiya Shinichi, Kurosaka Masahiro, Ouchi Kiyoshi, Kuroda Ryosuke, Mizuno Kosaku (2002). Graft Tension and Knee Stability After Anterior Cruciate Ligament Reconstruction. Clinical Orthopaedics and Related Research.

[14] Kawano CT, de Moraes Barros Fucs PM, Severino NR (2011). Pretensioning of quadruple flexor tendon grafts in two types of femoral fixation: quasi-randomised controlled pilot study. Int Orthop.

[15] Gong X, Jiang D, Wang YJ, Wang J, Ao YF, Yu JK (2013). Second-look arthroscopic evaluation of chondral lesions after isolated anterior cruciate ligament reconstruction: single- versus double-bundle reconstruction. Am J Sports Med.

[16] Harner Christopher D., Olson Eric, Irrgang James J., Silverstein Scott, Fu Freddie H., Silbey Mark (1996). Allograft Versus Autograft Anterior Cruciate Ligament Reconstruction. Clinical Orthopaedics and Related Research.

[17] Handl M, Drzik M, Cerulli G (2007). Reconstruction of the anterior cruciate ligament: dynamic strain evaluation of the graft. Knee Surg Sports Traumatol Arthrosc.

[18] Galbusera F, Freutel M, Durselen L (2014). Material models and properties in the finite element analysis of knee ligaments: a literature review. Front Bioeng Biotechnol.

[19] Brand J, Hamilton D, Selby J, Pienkowski D, Caborn DN, Johnson DL (2000). Biomechanical comparison of quadriceps tendon fixation with patellar tendon bone plug interference fixation in cruciate ligament reconstruction. Arthroscopy..

[20] Leiter JR, Gourlay R, McRae S, de Korompay N, MacDonald PB (2014). Long-term follow-up of ACL reconstruction with hamstring autograft. Knee Surg Sports Traumatol Arthrosc.

[21] Fu FH, Bennett CH, Lattermann C, Ma CB (1999). Current trends in anterior cruciate ligament reconstruction. Part 1: biology and biomechanics of reconstruction. Am J Sports Med.

[22] Hoher J, Scheffler SU, Withrow JD (2000). Mechanical behavior of two hamstring graft constructs for reconstruction of the anterior cruciate ligament. J Orthop Res.

[23] Heis FT, Paulos LE (2002). Tensioning of the anterior cruciate ligament graft. Orthop Clin North Am.

[24] Boylan D, Greis PE, West JR, Bachus KN, Burks RT (2003). Effects of initial graft tension on knee stability after anterior cruciate ligament reconstruction using hamstring tendons: a cadaver study. Arthroscopy..

[25] Arnold MP, Lie DT, Verdonschot N, de Graaf R, Amis AA, van Kampen A (2005). The remains of anterior cruciate ligament graft tension after cyclic knee motion. Am J Sports Med.

[26] Nurmi JT, Kannus P, Sievanen H, Jarvela T, Jarvinen M, Jarvinen TL (2004). Interference screw fixation of soft tissue grafts in anterior cruciate ligament reconstruction: part 2: effect of preconditioning on graft tension during and after screw insertion. Am J Sports Med.

[27] Nishizawa Y, Hoshino Y, Nagamune K (2017). Comparison between intra- and extra-articular tension of the graft during fixation in anterior cruciate ligament reconstruction. Arthroscopy..

[28] Noyes FR, Butler DL, Grood ES, Zernicke RF, Hefzy MS (1984). Biomechanical analysis of human ligament grafts used in knee-ligament repairs and reconstructions. J Bone Joint Surg Am.

[29] Whitehead TS (2013). Failure of anterior cruciate ligament reconstruction. Clin Sports Med.

[30] Sarraj M, de Sa D, Shanmugaraj A, Musahl V, Lesniak BP (2019). Over-the-top ACL reconstruction yields comparable outcomes to traditional ACL reconstruction in primary and revision settings: a systematic review. Knee Surg Sports Traumatol Arthrosc.

